# Exploration of biological network centralities with CentiBiN

**DOI:** 10.1186/1471-2105-7-219

**Published:** 2006-04-21

**Authors:** Björn H Junker, Dirk Koschützki, Falk Schreiber

**Affiliations:** 1 Department of Molecular Genetics, Leibniz Institute of Plant Genetics and Crop Plant Research (IPK), Corrensstr. 3, 06466 Gatersleben, Germany

## Abstract

**Background:**

The elucidation of whole-cell regulatory, metabolic, interaction and other biological networks generates the need for a meaningful ranking of network elements. *Centrality analysis *ranks network elements according to their importance within the network structure and different centrality measures focus on different importance concepts. Central elements of biological networks have been found to be, for example, essential for viability.

**Results:**

CentiBiN (Centralities in Biological Networks) is a tool for the computation and exploration of centralities in biological networks such as protein-protein interaction networks. It computes 17 different centralities for directed or undirected networks, ranging from local measures, that is, measures that only consider the direct neighbourhood of a network element, to global measures. CentiBiN supports the exploration of the centrality distribution by visualising central elements within the network and provides several layout mechanisms for the automatic generation of graphical representations of a network. It supports different input formats, especially for biological networks, and the export of the computed centralities to other tools.

**Conclusion:**

CentiBiN helps systems biology researchers to identify crucial elements of biological networks. CentiBiN including a user guide and example data sets is available free of charge at . CentiBiN is available in two different versions: a Java Web Start application and an installable Windows application.

## Background

The shift of biological research towards massively parallel techniques opens new opportunities but at the same time raises problems in deriving meaningful information out of the wealth of generated data. Such data might be represented as networks, in which the vertices (e.g. transcripts, proteins or metabolites) are linked by edges (correlations, interactions or reactions, respectively). Structural analysis of networks can lead to new insights into biological systems and is a helpful method for proposing new hypotheses. Several techniques for such structural analysis exist, such as the analysis of the global network structure (e.g. scale-free networks [1]), network motifs (i.e. small subnetworks which occur significantly more often in the biological network than in random networks [2]), network clustering (modularisation of the network into parts [3]) and network centralities [4]. Network centralities are used to rank elements of a network according to a given importance concept.

Ranking of network elements has been used to analyse biological networks in several cases. For example, it has been shown for metabolic networks that the most central metabolites are evolutionarily conserved [5]. Moreover, in the protein-protein interaction network of baker's yeast (*Saccharomyces cerevisiae*) it has been found that the centrality of a protein correlates with the essentiality of its gene, which was characterised by a high probability of a lethal effect observed upon knockout of this gene [6]. Recently, it has been observed that in transcript co-expression networks genes with high degree-centrality, that is, highly connected genes, tend to be essential and conserved [7].

**Figure 1:**
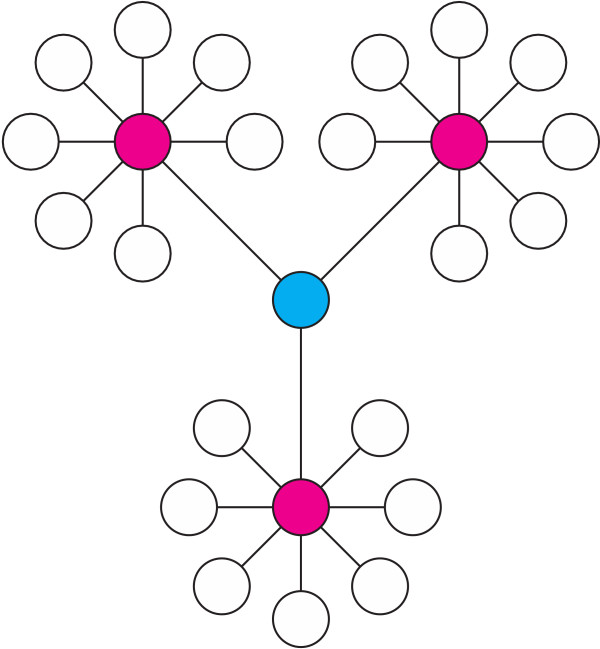
Different centrality measures rank vertices differently. The most important vertices according to the degree-centrality (red), that is, where a vertex of a network is central if it is highly connected, and the Closeness centrality (blue), that is, where a vertex is central if the sum of the shortest path distances to all the other vertices is small.

The determination of central elements in biological networks will create new hypotheses that lead to more rational approaches in experimental design. If the important elements of a network are known, further experimental investigations can be limited to them. Depending on the biological question, a vertex of a network might be of importance, for example, if it is connected to many other vertices or if the sum of the shortest path distances to all the other vertices is small, see Figure 1. For these different ranking concepts, a broad variety of centrality measures are available (see Table 1) which have been described in a recent review [8].

However, the use of centralities as a structural analysis method for biological networks is controversial and several centrality measures should be considered within an exploratory process [9]. To support such analysis and due to the complexity of both biological networks and centrality calculations, a tool is needed to facilitate these investigations. Here we present CentiBiN, an application for the calculation and visualisation of centralities for biological networks.

## Implementation

The core of CentiBiN are newly implemented algorithms for centrality analysis and visualisation (e.g. most of the centrality measures, the graphical user interface, cleanup methods and some imports/exports such as DOT, Pajek .vec and TSV). It is based on JUNG, the Java Universal Network/Graph Framework, an open source library which can be downloaded from [10]. JUNG provides standard graph library functionality (e.g. data structures, imports/exports, layouts, graph generators and a few centrality algorithms). CentiBiN is written in Java. It requires an installation of the Java Runtime Environment Version 1.4.2 or later which is available from the Java download page [11]. It is available free of charge as a Java Web Start application and an installable Windows application including a user guide and example data sets. Depending on available main memory and the centrality algorithm used networks up to several tens of thousands vertices can be analysed. Large networks with several thousand vertices are not readable on a computer screen anymore and the drawing routine significantly slows down for such networks. Thus they are not displayed, but can nevertheless be analysed and exported. A corresponding threshold is configurable by the user.

## Results and discussion

CentiBiN's major features are:

### Computation of centralities

CentiBiN supports a wide range of different centrality measures ranging from local measures (which only consider the direct neighbourhood of a vertex) to global measures. In total 17 centralities for undirected networks and 15 centralities for directed networks are available, see Table 2.

### Plotting the distribution of centrality values

**Figure 2:**
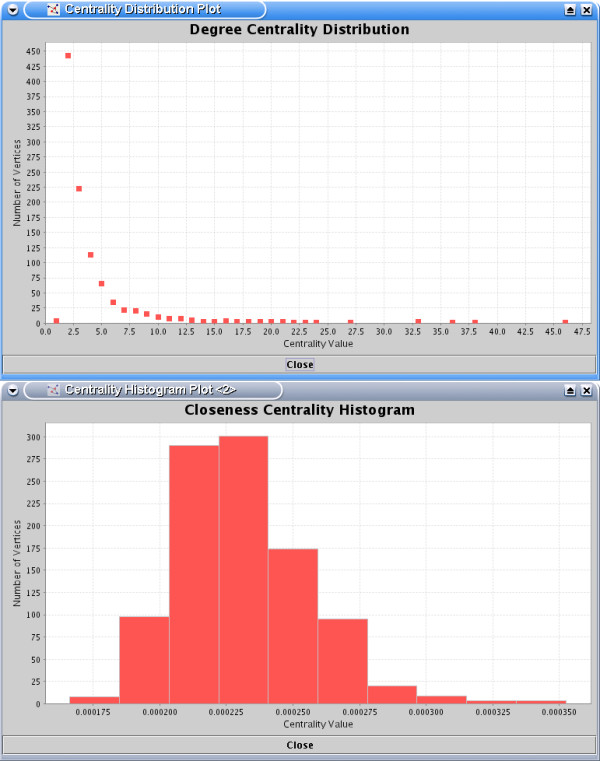
Plots generated with CentiBiN. The distribution of the degree centrality and a histogram of the closeness centrality for a random network.

The distribution of centrality values and a histogram of centrality values can be displayed, see Figure 2. Several of these diagrams can be opened simultaneously and allow the easy comparison of the centrality distributions.

### Visualisation and navigation within the network

From the list of centrality values several vertices can be selected. These are highlighted in the network and can therefore be easily located. Additionally CentiBiN supports zoom and pan functionalities to navigate within a displayed network. The underlying graph library offers five different layout algorithms for networks, reaching from simple circular to more advanced force directed layouts [12,13]. Depending on the network, one or the other layout method results in a better visualisation.

### Cleaning up a network

Depending on the centrality measure to be applied, the network has to fulfil certain preconditions. These can be simplicity, connectedness, and loop-freeness. Therefore, several algorithms are implemented for transforming a network into the required form. These are the removal of all loops (edges from one vertex to itself), the removal of all vertices that are not part of the largest connected component (giant strong component), the removal of all parallel edges, and the transformation of the network into an undirected or directed form.

### Reading and writing networks and centralities

Networks can be loaded out of four different file formats: the Pajek .net file format [14], a text file containing an adjacency matrix representation of a network, the GraphML file format [15] and the TSV-files provided by the Database of Interacting Proteins (DIP) [16]. It is possible to store networks in the Pajek .net file format, in a text file containing a representation of the adjacency matrix, and in the DOT file format used by Graphviz [17]. To support further analysis of computed centralities they can be saved either in the Pajek .vec file format or in a TSV format. These files can be imported in other applications, such as R [18].

### Generation of random networks

The generation of random networks based on five different algorithms (provided by JUNG), such as Kleinberg's small-world generator [19] and the Barabási-Albert scale-free generator [20], is available. These networks can be analysed and visualised and may be used as reference models.

### Analysing protein-protein interactions

**Figure 3:**
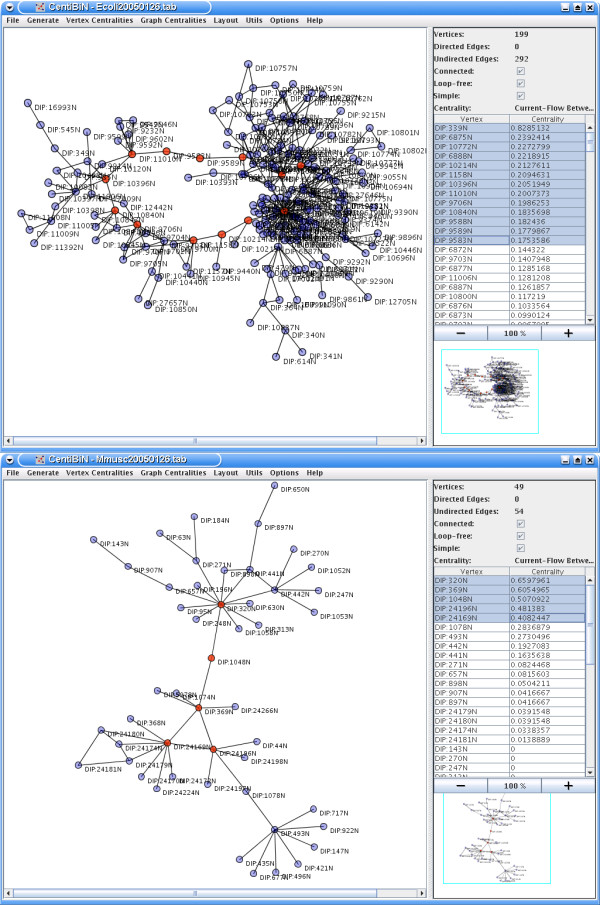
Two screen-shots showing centrality analysis with CentiBiN. The networks represent protein-protein interactions in *Escherichia coli *(top) and *Mus musculus *(bottom) according to the Database of Interacting Proteins (DIP), release 2005-01-26. The top ranking proteins according to the Current-Flow Betweenness centrality [21] are highlighted.

A typical example for networks which can be analysed are protein-protein interaction networks. All interactions between proteins of an organism can be represented as a network. Several databases contain information about such protein-protein interactions. We chose the Database of Interacting Proteins (DIP) [16] from which interaction data can be imported into CentiBiN. Figure 3 shows screen-shots of the tool with interaction data from *Escherichia coli *and *Mus musculus *(mouse). The most import proteins according to the Current-Flow betweenness [21] are highlighted.

### Comparison and outlook

Besides CentiBiN, several other software tools support the analysis of networks with centralities. Many of them are described in detail in the work of Huisman and van Duijn [22]. The focus of their comparison lies on software for social network analysis, an area where interactions between individuals are analysed. As the original concept of centralities can be traced back into this field of science, software packages for social network analysis often provide methods for centrality analysis. Some of the tool sevaluated by Huisman and van Duijn are commercial (UCINET, NetMiner), have a text based front end (STRUCTURE) or are software systems for advanced statistical modelling of social networks (StOCNet). Furthermore, only some of them have more than a few centralities implemented (e.g. MultiNet, Visone, Pajek). Most of the tools specifically designed for the analysis of biological networks (e.g. Cytoscape [23], Osprey [24]) do not support centrality analysis so far. The distribution plot (see Figure 2) is similar to the plot available in VisANT [25]. Compared to CentiBiN none of the available systems covers this extensive number of different centrality measures. Moreover, CentiBiN supports direct access to biological data, allows the visualisation of the network and the centralities together, has a simple input file format, and is available free of charge. For the next release we plan to integrate several methods for comparison of centralities with biological information. For that it is foreseen to integrate mechanisms to mark vertices according to given information or to correlate centralities with experimental results. Additionally we plan to support more input formats, for example PSI's Molecular Interaction XML Format [26] and to implement advanced visualisation methods.

## Conclusion

CentiBiN is a tool for the computation and exploration of *centralities in biological networks*. With CentiBiN it is possible to infer information about the "importance" of an element in a biological network based on different importance concepts. We have applied this for protein-protein interaction networks. We are convinced that CentiBiN provides valuable help to systems biologists in the generation of hypotheses from large-scale data sets.

## Availability and requirements

• **Project name: **CentiBiN

• **Project home page: **

• **Operating system (s): **Platform independent

• **Programming language: **Java

• **Other requirements: **Java 1.4.2 or higher, 256 MB RAM recommended

• **License: **The CentiBiN application is available free of charge.

• **Any restrictions to use by non-academics: **none

## Authors' contributions

All authors participated in the design of the system. DK implemented the system. DK and BHJ drafted the manuscript. All authors read, revised and approved the final version and were listed in alphabetical order.

## Figures and Tables

**Table 1:** Definitions for the centrality measures. Let *G *= (*V*, *E *be an undirected or directed, (strong) connected graph with *n *= |*V*| vertices; deg(*v*) denotes the degree of the vertex *v *in an undirected graph; dist(*v*, *w*) denotes the length of a shortest path between the vertices *s *and *t*; *σ*_*st *_denotes the number of shortest paths from *s *to *t *and *σ*_*st*_(*v*) the number of shortest path from *s *to *t *that use the vertex *v*. Let *A *be the adjacency matrix of the graph *G*. For a more detailed description and further references please see [8, 27]. Abbreviations used: S.-P.: shortest path, C.-F.: current flow.

**Name**	**Definition**	**Remarks**	**Ref**
Degree	C MathType@MTEF@5@5@+=feaafiart1ev1aaatCvAUfKttLearuWrP9MDH5MBPbIqV92AaeXatLxBI9gBamrtHrhAL1wy0L2yHvtyaeHbnfgDOvwBHrxAJfwnaebbnrfifHhDYfgasaacH8akY=wiFfYdH8Gipec8Eeeu0xXdbba9frFj0=OqFfea0dXdd9vqai=hGuQ8kuc9pgc9s8qqaq=dirpe0xb9q8qiLsFr0=vr0=vr0dc8meaabaqaciaacaGaaeqabaWaaeGaeaaakeaaimaacqWFce=qaaa@3825@_*deg*_(*v*) := deg(*v*)	For directed graphs in- and out-degree is used.	
Eccentricity	Cℯcc(v):=1max⁡{dist(v,w):w∈V} MathType@MTEF@5@5@+=feaafiart1ev1aaatCvAUfKttLearuWrP9MDH5MBPbIqV92AaeXatLxBI9gBamrtHrhAL1wy0L2yHvtyaeHbnfgDOvwBHrxAJfwnaebbnrfifHhDYfgasaacH8akY=wiFfYdH8Gipec8Eeeu0xXdbba9frFj0=OqFfea0dXdd9vqai=hGuQ8kuc9pgc9s8qqaq=dirpe0xb9q8qiLsFr0=vr0=vr0dc8meaabaqaciaacaGaaeqabaWaaeGaeaaakeaaimaacqWFce=qdaWgaaWcbaGae83lHuMae8hhXpMae8hhXpgabeaakmaabmaabaGaemODayhacaGLOaGaayzkaaGaeiOoaOJaeyypa0ZaaSaaaeaacqaIXaqmaeaacyGGTbqBcqGGHbqycqGG4baEdaGadaqaaiabbsgaKjabbMgaPjabbohaZjabbsha0naabmaabaGaemODayNaeiilaWIaem4DaChacaGLOaGaayzkaaGaeiOoaOJaem4DaCNaeyicI4SaemOvayfacaGL7bGaayzFaaaaaaaa@59DB@		[28]
Closeness	Ccℓℴ(v):=1∑w∈Vdist(v,w) MathType@MTEF@5@5@+=feaafiart1ev1aaatCvAUfKttLearuWrP9MDH5MBPbIqV92AaeXatLxBI9gBamrtHrhAL1wy0L2yHvtyaeHbnfgDOvwBHrxAJfwnaebbnrfifHhDYfgasaacH8akY=wiFfYdH8Gipec8Eeeu0xXdbba9frFj0=OqFfea0dXdd9vqai=hGuQ8kuc9pgc9s8qqaq=dirpe0xb9q8qiLsFr0=vr0=vr0dc8meaabaqaciaacaGaaeqabaWaaeGaeaaakeaaimaacqWFce=qdaWgaaWcbaGae8hhXpMae83eHWMae8hnH8gabeaakmaabmaabaGaemODayhacaGLOaGaayzkaaGaeiOoaOJaeyypa0ZaaSaaaeaacqaIXaqmaeaadaaeqaqaaiabbsgaKjabbMgaPjabbohaZjabbsha0naabmaabaGaemODayNaeiilaWIaem4DaChacaGLOaGaayzkaaaaleaacqWG3bWDcqGHiiIZcqWGwbGvaeqaniabggHiLdaaaaaa@53AA@		[29]
Radiality	Crad(v):=∑w∈V(ΔG+1−dist(v,w))n−1 MathType@MTEF@5@5@+=feaafiart1ev1aaatCvAUfKttLearuWrP9MDH5MBPbIqV92AaeXatLxBI9gBamrtHrhAL1wy0L2yHvtyaeHbnfgDOvwBHrxAJfwnaebbnrfifHhDYfgasaacH8akY=wiFfYdH8Gipec8Eeeu0xXdbba9frFj0=OqFfea0dXdd9vqai=hGuQ8kuc9pgc9s8qqaq=dirpe0xb9q8qiLsFr0=vr0=vr0dc8meaabaqaciaacaGaaeqabaWaaeGaeaaakeaaimaacqWFce=qdaWgaaWcbaGae83kXlNae8NgXhMae8xhXtgabeaakmaabmaabaGaemODayhacaGLOaGaayzkaaGaeiOoaOJaeyypa0ZaaSaaaeaadaaeqaqaamaabmaabaGaeuiLdq0aaSbaaSqaaGqaciab+DeahbqabaGccqGHRaWkcqaIXaqmcqGHsislcqqGKbazcqqGPbqAcqqGZbWCcqqG0baDdaqadaqaaiabdAha2jabcYcaSiabdEha3bGaayjkaiaawMcaaaGaayjkaiaawMcaaaWcbaGaem4DaCNaeyicI4SaemOvayfabeqdcqGHris5aaGcbaGaemOBa4MaeyOeI0IaeGymaedaaaaa@5E9F@	Δ_*G *_is the diameter of the graph *G*, defined as the maximum distance between any two vertices of *G*.	[30]
Centroid Value	C MathType@MTEF@5@5@+=feaafiart1ev1aaatCvAUfKttLearuWrP9MDH5MBPbIqV92AaeXatLxBI9gBamrtHrhAL1wy0L2yHvtyaeHbnfgDOvwBHrxAJfwnaebbnrfifHhDYfgasaacH8akY=wiFfYdH8Gipec8Eeeu0xXdbba9frFj0=OqFfea0dXdd9vqai=hGuQ8kuc9pgc9s8qqaq=dirpe0xb9q8qiLsFr0=vr0=vr0dc8meaabaqaciaacaGaaeqabaWaaeGaeaaakeaaimaacqWFce=qaaa@3825@_*cen*_(*v*) := min{f(*v*, *w*) : *w *∈ *V*\{*v*}}	Where *f*(*v*, *w*) := *γ*_*v*_(*w*) - *γ*_*w*_(*v*) and *γ*_*v*_(*w*) denotes the number of vertices that are closer to *v *than to *w*.	[31]
Stress	C MathType@MTEF@5@5@+=feaafiart1ev1aaatCvAUfKttLearuWrP9MDH5MBPbIqV92AaeXatLxBI9gBamrtHrhAL1wy0L2yHvtyaeHbnfgDOvwBHrxAJfwnaebbnrfifHhDYfgasaacH8akY=wiFfYdH8Gipec8Eeeu0xXdbba9frFj0=OqFfea0dXdd9vqai=hGuQ8kuc9pgc9s8qqaq=dirpe0xb9q8qiLsFr0=vr0=vr0dc8meaabaqaciaacaGaaeqabaWaaeGaeaaakeaaimaacqWFce=qaaa@3825@_*str *_(*v*) :=∑_*s*≠*v*∈*V *_∑_*t*≠*v*∈*V *_*σ*_*st*_(*v*)		[32]
S.-P. Betweenness	C MathType@MTEF@5@5@+=feaafiart1ev1aaatCvAUfKttLearuWrP9MDH5MBPbIqV92AaeXatLxBI9gBamrtHrhAL1wy0L2yHvtyaeHbnfgDOvwBHrxAJfwnaebbnrfifHhDYfgasaacH8akY=wiFfYdH8Gipec8Eeeu0xXdbba9frFj0=OqFfea0dXdd9vqai=hGuQ8kuc9pgc9s8qqaq=dirpe0xb9q8qiLsFr0=vr0=vr0dc8meaabaqaciaacaGaaeqabaWaaeGaeaaakeaaimaacqWFce=qaaa@3825@_*spb *_(*v*) :=∑_*s*≠*v*∈*V *_∑_*t*≠*v*∈*V *_*σ*_*st*_(*v*)	δst(v):=σst(v)σst MathType@MTEF@5@5@+=feaafiart1ev1aaatCvAUfKttLearuWrP9MDH5MBPbIqV92AaeXatLxBI9gBaebbnrfifHhDYfgasaacH8akY=wiFfYdH8Gipec8Eeeu0xXdbba9frFj0=OqFfea0dXdd9vqai=hGuQ8kuc9pgc9s8qqaq=dirpe0xb9q8qiLsFr0=vr0=vr0dc8meaabaqaciaacaGaaeqabaqabeGadaaakeaaiiGacqWF0oazdaWgaaWcbaGaem4CamNaemiDaqhabeaakmaabmaabaGaemODayhacaGLOaGaayzkaaGaeiOoaOJaeyypa0ZaaSaaaeaacqWFdpWCdaWgaaWcbaGaem4CamNaemiDaqhabeaakmaabmaabaGaemODayhacaGLOaGaayzkaaaabaGae83Wdm3aaSbaaSqaaiabdohaZjabdsha0bqabaaaaaaa@431A@	[33]
C.-F. Closeness	Ccfc(v)=n−1∑t∉vpvt(v)−pvt(t) MathType@MTEF@5@5@+=feaafiart1ev1aaatCvAUfKttLearuWrP9MDH5MBPbIqV92AaeXatLxBI9gBamrtHrhAL1wy0L2yHvtyaeHbnfgDOvwBHrxAJfwnaebbnrfifHhDYfgasaacH8akY=wiFfYdH8Gipec8Eeeu0xXdbba9frFj0=OqFfea0dXdd9vqai=hGuQ8kuc9pgc9s8qqaq=dirpe0xb9q8qiLsFr0=vr0=vr0dc8meaabaqaciaacaGaaeqabaWaaeGaeaaakeaaimaacqWFce=qdaWgaaWcbaGae8hhXpMae83hXBMae8hhXpgabeaakmaabmaabaGaemODayhacaGLOaGaayzkaaGaeyypa0ZaaSaaaeaacqWGUbGBcqGHsislcqaIXaqmaeaadaaeqaqaaiabdchaWnaaBaaaleaacqWG2bGDcqWG0baDaeqaaOWaaeWaaeaacqWG2bGDaiaawIcacaGLPaaacqGHsislcqWGWbaCdaWgaaWcbaGaemODayNaemiDaqhabeaakmaabmaabaGaemiDaqhacaGLOaGaayzkaaaaleaacqWG0baDcqGHjiYZcqWG2bGDaeqaniabggHiLdaaaaaa@5BD4@	Where *p*_*vt*_(*t*) equals the potential difference in an electrical network.	[21]
C.-F. Betweenness	Ccfb(v)=1(n−1)(n−2)∑s,t∈Vτst(v) MathType@MTEF@5@5@+=feaafiart1ev1aaatCvAUfKttLearuWrP9MDH5MBPbIqV92AaeXatLxBI9gBamrtHrhAL1wy0L2yHvtyaeHbnfgDOvwBHrxAJfwnaebbnrfifHhDYfgasaacH8akY=wiFfYdH8Gipec8Eeeu0xXdbba9frFj0=OqFfea0dXdd9vqai=hGuQ8kuc9pgc9s8qqaq=dirpe0xb9q8qiLsFr0=vr0=vr0dc8meaabaqaciaacaGaaeqabaWaaeGaeaaakeaaimaacqWFce=qdaWgaaWcbaGae8hhXpMae83hXBMae83gXlgabeaakmaabmaabaGaemODayhacaGLOaGaayzkaaGaeyypa0ZaaSaaaeaacqaIXaqmaeaadaqadaqaaiabd6gaUjabgkHiTiabigdaXaGaayjkaiaawMcaamaabmaabaGaemOBa4MaeyOeI0IaeGOmaidacaGLOaGaayzkaaaaamaaqababaacciGae4hXdq3aaSbaaSqaaiabdohaZjabdsha0bqabaGcdaqadaqaaiabdAha2bGaayjkaiaawMcaaaWcbaGaem4CamNaeiilaWIaemiDaqNaeyicI4SaemOvayfabeqdcqGHris5aaaa@5D15@	Where *τ*_*st*_(*v*) equals the fraction of electrical current running over vertex *v *in an electrical network.	[21]
Katz Status	Ckatz:=∑k=1∞αk(AT)k1→ MathType@MTEF@5@5@+=feaafiart1ev1aaatCvAUfKttLearuWrP9MDH5MBPbIqV92AaeXatLxBI9gBamrtHrhAL1wy0L2yHvtyaeHbnfgDOvwBHrxAJfwnaebbnrfifHhDYfgasaacH8akY=wiFfYdH8Gipec8Eeeu0xXdbba9frFj0=OqFfea0dXdd9vqai=hGuQ8kuc9pgc9s8qqaq=dirpe0xb9q8qiLsFr0=vr0=vr0dc8meaabaqaciaacaGaaeqabaWaaeGaeaaakeaaimaacqWFce=qdaWgaaWcbaGae8hjXVMae8NgXhMae8xlXtNae83mXRhabeaakiabcQda6iabg2da9maaqadabaacciGae4xSde2aaWbaaSqabeaacqWGRbWAaaGcdaqadaqaaiabdgeabnaaCaaaleqabaGaemivaqfaaaGccaGLOaGaayzkaaWaaWbaaSqabeaacqWGRbWAaaGccuaIXaqmgaWcaaWcbaGaem4AaSMaeyypa0JaeGymaedabaGae4NhIukaniabggHiLdaaaa@531C@	Where *α *is a positive constant.	[34]
Eigenvector	*λ*C MathType@MTEF@5@5@+=feaafiart1ev1aaatCvAUfKttLearuWrP9MDH5MBPbIqV92AaeXatLxBI9gBamrtHrhAL1wy0L2yHvtyaeHbnfgDOvwBHrxAJfwnaebbnrfifHhDYfgasaacH8akY=wiFfYdH8Gipec8Eeeu0xXdbba9frFj0=OqFfea0dXdd9vqai=hGuQ8kuc9pgc9s8qqaq=dirpe0xb9q8qiLsFr0=vr0=vr0dc8meaabaqaciaacaGaaeqabaWaaeGaeaaakeaaimaacqWFce=qaaa@3825@_*eiv *_= *A*C MathType@MTEF@5@5@+=feaafiart1ev1aaatCvAUfKttLearuWrP9MDH5MBPbIqV92AaeXatLxBI9gBamrtHrhAL1wy0L2yHvtyaeHbnfgDOvwBHrxAJfwnaebbnrfifHhDYfgasaacH8akY=wiFfYdH8Gipec8Eeeu0xXdbba9frFj0=OqFfea0dXdd9vqai=hGuQ8kuc9pgc9s8qqaq=dirpe0xb9q8qiLsFr0=vr0=vr0dc8meaabaqaciaacaGaaeqabaWaaeGaeaaakeaaimaacqWFce=qaaa@3825@_*eiv*_	The eigenvector to the dominant eigenvalue of *A *is used.	[35]
Hubbell index	C MathType@MTEF@5@5@+=feaafiart1ev1aaatCvAUfKttLearuWrP9MDH5MBPbIqV92AaeXatLxBI9gBamrtHrhAL1wy0L2yHvtyaeHbnfgDOvwBHrxAJfwnaebbnrfifHhDYfgasaacH8akY=wiFfYdH8Gipec8Eeeu0xXdbba9frFj0=OqFfea0dXdd9vqai=hGuQ8kuc9pgc9s8qqaq=dirpe0xb9q8qiLsFr0=vr0=vr0dc8meaabaqaciaacaGaaeqabaWaaeGaeaaakeaaimaacqWFce=qaaa@3825@_*hbl *_= E→ MathType@MTEF@5@5@+=feaafiart1ev1aaatCvAUfKttLearuWrP9MDH5MBPbIqV92AaeXatLxBI9gBaebbnrfifHhDYfgasaacH8akY=wiFfYdH8Gipec8Eeeu0xXdbba9frFj0=OqFfea0dXdd9vqai=hGuQ8kuc9pgc9s8qqaq=dirpe0xb9q8qiLsFr0=vr0=vr0dc8meaabaqaciaacaGaaeqabaqabeGadaaakeaacuWGfbqrgaWcaaaa@2DD1@ + *W*C MathType@MTEF@5@5@+=feaafiart1ev1aaatCvAUfKttLearuWrP9MDH5MBPbIqV92AaeXatLxBI9gBamrtHrhAL1wy0L2yHvtyaeHbnfgDOvwBHrxAJfwnaebbnrfifHhDYfgasaacH8akY=wiFfYdH8Gipec8Eeeu0xXdbba9frFj0=OqFfea0dXdd9vqai=hGuQ8kuc9pgc9s8qqaq=dirpe0xb9q8qiLsFr0=vr0=vr0dc8meaabaqaciaacaGaaeqabaWaaeGaeaaakeaaimaacqWFce=qaaa@3825@_*hbl*_	Where E→ MathType@MTEF@5@5@+=feaafiart1ev1aaatCvAUfKttLearuWrP9MDH5MBPbIqV92AaeXatLxBI9gBaebbnrfifHhDYfgasaacH8akY=wiFfYdH8Gipec8Eeeu0xXdbba9frFj0=OqFfea0dXdd9vqai=hGuQ8kuc9pgc9s8qqaq=dirpe0xb9q8qiLsFr0=vr0=vr0dc8meaabaqaciaacaGaaeqabaqabeGadaaakeaacuWGfbqrgaWcaaaa@2DD1@ is some exogenous input and *W *is a weight matrix derived from the adjacency matrix *A*.	[36]
Bargaining	C MathType@MTEF@5@5@+=feaafiart1ev1aaatCvAUfKttLearuWrP9MDH5MBPbIqV92AaeXatLxBI9gBamrtHrhAL1wy0L2yHvtyaeHbnfgDOvwBHrxAJfwnaebbnrfifHhDYfgasaacH8akY=wiFfYdH8Gipec8Eeeu0xXdbba9frFj0=OqFfea0dXdd9vqai=hGuQ8kuc9pgc9s8qqaq=dirpe0xb9q8qiLsFr0=vr0=vr0dc8meaabaqaciaacaGaaeqabaWaaeGaeaaakeaaimaacqWFce=qaaa@3825@_*brg *_:= *α*(*I *- *βA*)^-1 ^*A*1→ MathType@MTEF@5@5@+=feaafiart1ev1aaatCvAUfKttLearuWrP9MDH5MBPbIqV92AaeXatLxBI9gBaebbnrfifHhDYfgasaacH8akY=wiFfYdH8Gipec8Eeeu0xXdbba9frFj0=OqFfea0dXdd9vqai=hGuQ8kuc9pgc9s8qqaq=dirpe0xb9q8qiLsFr0=vr0=vr0dc8meaabaqaciaacaGaaeqabaqabeGadaaakeaacuaIXaqmgaWcaaaa@2DAE@	Where a is *α *scaling factor and *β *is the influence parameter.	[37]
PageRank	C MathType@MTEF@5@5@+=feaafiart1ev1aaatCvAUfKttLearuWrP9MDH5MBPbIqV92AaeXatLxBI9gBamrtHrhAL1wy0L2yHvtyaeHbnfgDOvwBHrxAJfwnaebbnrfifHhDYfgasaacH8akY=wiFfYdH8Gipec8Eeeu0xXdbba9frFj0=OqFfea0dXdd9vqai=hGuQ8kuc9pgc9s8qqaq=dirpe0xb9q8qiLsFr0=vr0=vr0dc8meaabaqaciaacaGaaeqabaWaaeGaeaaakeaaimaacqWFce=qaaa@3825@_*pr *_= *dP*C MathType@MTEF@5@5@+=feaafiart1ev1aaatCvAUfKttLearuWrP9MDH5MBPbIqV92AaeXatLxBI9gBamrtHrhAL1wy0L2yHvtyaeHbnfgDOvwBHrxAJfwnaebbnrfifHhDYfgasaacH8akY=wiFfYdH8Gipec8Eeeu0xXdbba9frFj0=OqFfea0dXdd9vqai=hGuQ8kuc9pgc9s8qqaq=dirpe0xb9q8qiLsFr0=vr0=vr0dc8meaabaqaciaacaGaaeqabaWaaeGaeaaakeaaimaacqWFce=qaaa@3825@_*pr *_+ (1 - *d*)1→ MathType@MTEF@5@5@+=feaafiart1ev1aaatCvAUfKttLearuWrP9MDH5MBPbIqV92AaeXatLxBI9gBaebbnrfifHhDYfgasaacH8akY=wiFfYdH8Gipec8Eeeu0xXdbba9frFj0=OqFfea0dXdd9vqai=hGuQ8kuc9pgc9s8qqaq=dirpe0xb9q8qiLsFr0=vr0=vr0dc8meaabaqaciaacaGaaeqabaqabeGadaaakeaacuaIXaqmgaWcaaaa@2DAE@	Where *P *is the transition matrix and *d *is the damping factor.	[38]
HITS-Hubs	C MathType@MTEF@5@5@+=feaafiart1ev1aaatCvAUfKttLearuWrP9MDH5MBPbIqV92AaeXatLxBI9gBamrtHrhAL1wy0L2yHvtyaeHbnfgDOvwBHrxAJfwnaebbnrfifHhDYfgasaacH8akY=wiFfYdH8Gipec8Eeeu0xXdbba9frFj0=OqFfea0dXdd9vqai=hGuQ8kuc9pgc9s8qqaq=dirpe0xb9q8qiLsFr0=vr0=vr0dc8meaabaqaciaacaGaaeqabaWaaeGaeaaakeaaimaacqWFce=qaaa@3825@_*hubs *_= *A*C MathType@MTEF@5@5@+=feaafiart1ev1aaatCvAUfKttLearuWrP9MDH5MBPbIqV92AaeXatLxBI9gBamrtHrhAL1wy0L2yHvtyaeHbnfgDOvwBHrxAJfwnaebbnrfifHhDYfgasaacH8akY=wiFfYdH8Gipec8Eeeu0xXdbba9frFj0=OqFfea0dXdd9vqai=hGuQ8kuc9pgc9s8qqaq=dirpe0xb9q8qiLsFr0=vr0=vr0dc8meaabaqaciaacaGaaeqabaWaaeGaeaaakeaaimaacqWFce=qaaa@3825@_*auths*_	Assuming C MathType@MTEF@5@5@+=feaafiart1ev1aaatCvAUfKttLearuWrP9MDH5MBPbIqV92AaeXatLxBI9gBamrtHrhAL1wy0L2yHvtyaeHbnfgDOvwBHrxAJfwnaebbnrfifHhDYfgasaacH8akY=wiFfYdH8Gipec8Eeeu0xXdbba9frFj0=OqFfea0dXdd9vqai=hGuQ8kuc9pgc9s8qqaq=dirpe0xb9q8qiLsFr0=vr0=vr0dc8meaabaqaciaacaGaaeqabaWaaeGaeaaakeaaimaacqWFce=qaaa@3825@_*auths *_is known.	[39]
HITS-Authorities	C MathType@MTEF@5@5@+=feaafiart1ev1aaatCvAUfKttLearuWrP9MDH5MBPbIqV92AaeXatLxBI9gBamrtHrhAL1wy0L2yHvtyaeHbnfgDOvwBHrxAJfwnaebbnrfifHhDYfgasaacH8akY=wiFfYdH8Gipec8Eeeu0xXdbba9frFj0=OqFfea0dXdd9vqai=hGuQ8kuc9pgc9s8qqaq=dirpe0xb9q8qiLsFr0=vr0=vr0dc8meaabaqaciaacaGaaeqabaWaaeGaeaaakeaaimaacqWFce=qaaa@3825@_*auths *_= *A*^*T*^C MathType@MTEF@5@5@+=feaafiart1ev1aaatCvAUfKttLearuWrP9MDH5MBPbIqV92AaeXatLxBI9gBamrtHrhAL1wy0L2yHvtyaeHbnfgDOvwBHrxAJfwnaebbnrfifHhDYfgasaacH8akY=wiFfYdH8Gipec8Eeeu0xXdbba9frFj0=OqFfea0dXdd9vqai=hGuQ8kuc9pgc9s8qqaq=dirpe0xb9q8qiLsFr0=vr0=vr0dc8meaabaqaciaacaGaaeqabaWaaeGaeaaakeaaimaacqWFce=qaaa@3825@_*hubs*_	Assuming C MathType@MTEF@5@5@+=feaafiart1ev1aaatCvAUfKttLearuWrP9MDH5MBPbIqV92AaeXatLxBI9gBamrtHrhAL1wy0L2yHvtyaeHbnfgDOvwBHrxAJfwnaebbnrfifHhDYfgasaacH8akY=wiFfYdH8Gipec8Eeeu0xXdbba9frFj0=OqFfea0dXdd9vqai=hGuQ8kuc9pgc9s8qqaq=dirpe0xb9q8qiLsFr0=vr0=vr0dc8meaabaqaciaacaGaaeqabaWaaeGaeaaakeaaimaacqWFce=qaaa@3825@_*hubs *_is known.	[39]
Closeness- vitality	C MathType@MTEF@5@5@+=feaafiart1ev1aaatCvAUfKttLearuWrP9MDH5MBPbIqV92AaeXatLxBI9gBamrtHrhAL1wy0L2yHvtyaeHbnfgDOvwBHrxAJfwnaebbnrfifHhDYfgasaacH8akY=wiFfYdH8Gipec8Eeeu0xXdbba9frFj0=OqFfea0dXdd9vqai=hGuQ8kuc9pgc9s8qqaq=dirpe0xb9q8qiLsFr0=vr0=vr0dc8meaabaqaciaacaGaaeqabaWaaeGaeaaakeaaimaacqWFce=qaaa@3825@_*clv*_(*v*) := WI(*G*) - WI(*G*\{*v*})	Where WI(*G*) is the Wiener index of the graph *G*.	[8]

**Table 2:** Centrality measures implemented in CentiBiN. Definitions for these measures can be found in Table 1.

Centrality	Type	Directed graphs	Undirected graphs
Degree	Neighbourhood based	Yes^1^	Yes
Eccentricity	Distance based	Yes	Yes
Closeness	Distance based	Yes	Yes
Radiality	Distance based	Yes	Yes
Centroid	Distance based	Yes	Yes
Stress	Shortest-Path based	Yes	Yes
S.-P. Betweenness	Shortest-Path based	Yes	Yes
C.-F. Betweenness	Current-Flow based	No^2^	Yes
C.-F. Closeness	Current-Flow based	No^2^	Yes
Katz Status	Feedback based	Yes	Yes
Eigenvector	Feedback based	Yes	Yes
Hubbell index	Feedback based	Yes	Yes
Bargaining	Feedback based	Yes	Yes
PageRank	Feedback based	Yes	Yes
HITS-Hubs	Feedback based	Yes	Yes^3^
HITS-Auths	Feedback based	Yes	Yes^3^
Closeness- vitality	Vitality based	Yes	Yes

^1 ^both in- and out-degree

^2 ^not defined for directed graphs

^3 ^HITS-Hubs and HITS-Auths give identical results for undirected graphs
